# Assessing the Spectral Properties of Sunlit and Shaded Components in Rice Canopies with Near-Ground Imaging Spectroscopy Data

**DOI:** 10.3390/s17030578

**Published:** 2017-03-13

**Authors:** Kai Zhou, Xinqiang Deng, Xia Yao, Yongchao Tian, Weixing Cao, Yan Zhu, Susan L. Ustin, Tao Cheng

**Affiliations:** 1 National Engineering and Technology Center for Information Agriculture, Jiangsu Key Laboratory for Information Agriculture, Jiangsu Collaborative Innovation Center for Modern Crop Production, Nanjing Agricultural University, Nanjing 210095, China; 2013201073@njau.edu.cn (K.Z.); 2013101011@njau.edu.cn (X.D.); yaoxia@njau.edu.cn (X.Y.); yctian@njau.edu.cn (Y.T.); caow@njau.edu.cn (W.C.); 2 Center for Spatial Technologies and Remote Sensing (CSTARS), Department of Land, Air, and Water Resources, University of California, Davis, CA 95616-8617, USA; slustin@ucdavis.edu

**Keywords:** shadow, hyperspectral, rice leaf, rice panicle, red edge, spectral index, chlorophyll content

## Abstract

Monitoring the components of crop canopies with remote sensing can help us understand the within-canopy variation in spectral properties and resolve the sources of uncertainties in the spectroscopic estimation of crop foliar chemistry. To date, the spectral properties of leaves and panicles in crop canopies and the shadow effects on their spectral variation remain poorly understood due to the insufficient spatial resolution of traditional spectroscopy data. To address this issue, we used a near-ground imaging spectroscopy system with high spatial and spectral resolutions to examine the spectral properties of rice leaves and panicles in sunlit and shaded portions of canopies and evaluate the effect of shadows on the relationships between spectral indices of leaves and foliar chlorophyll content. The results demonstrated that the shaded components exhibited lower reflectance amplitude but stronger absorption features than their sunlit counterparts. Specifically, the reflectance spectra of panicles had unique double-peak absorption features in the blue region. Among the examined vegetation indices (VIs), significant differences were found in the photochemical reflectance index (PRI) between leaves and panicles and further differences in the transformed chlorophyll absorption reflectance index (TCARI) between sunlit and shaded components. After an image-level separation of canopy components with these two indices, statistical analyses revealed much higher correlations between canopy chlorophyll content and both PRI and TCARI of shaded leaves than for those of sunlit leaves. In contrast, the red edge chlorophyll index (CI_Red-edge_) exhibited the strongest correlations with canopy chlorophyll content among all vegetation indices examined regardless of shadows on leaves. These findings represent significant advances in the understanding of rice leaf and panicle spectral properties under natural light conditions and demonstrate the significance of commonly overlooked shaded leaves in the canopy when correlated to canopy chlorophyll content.

## 1. Introduction

A crop canopy under natural light conditions is composed of sunlit and shaded parts, as shadows arise from blocking a fraction of direct light from solar illumination [1]. Although shadows typically appear dark on visualized images, shaded foliage contributes to crop biophysical signals carried through diffuse irradiance from within the canopy and represents a significant contributor to canopy reflectance properties [2,3,4]. In the community of vegetation mapping, most studies focus on the sunlit portions of the upper canopy and discard the shaded portions in subsequent spectral analysis [5,6,7,8,9,10,11]. Others assume that the reflectance values of shadows equal zero or are constant for the purposes of spectral mixture analysis [2,12] and estimation of canopy parameters with radiative transfer [13,14] or geometrical optical models [15,16,17]. These studies suggest that the potential spectral information in shaded portions of the canopy have not been fully exploited.

Nevertheless, recent studies have demonstrated differences between sunlit leaves (SL) and shaded leaves (SHL) of crops in the monitoring of sun-induced chlorophyll fluorescence [18,19], vegetation photosynthesis and light use efficiency (LUE) [20,21]. These relevant studies were devoted to such crops as sugar beet, barley, corn and wheat and focused on observations of leaves only. Other components in the canopy, such as rice panicles that emerge at advanced reproductive stages while coexisting with leaves and may be even more exposed to instruments observing from the top of the canopy, were not considered in these field experiments. The presence of panicles together with sunlit and shaded portions in rice canopies create uncertainties in the quantification of crop chemistry from canopy reflectance spectra, and it remains unclear how to cope with the coexistence of leaves and panicles in sunlit and shaded forms with traditional spectroscopy technologies.

For monitoring rice growth status, many studies have used traditional non-imaging spectrometers to collect reflectance spectra at canopy level [22,23]. Those canopy-level spectral data become inappropriate for fine scale precision farming [24,25], because non-imaging spectrometers operated above the canopy cannot record the spectral reflectance of individual organs such as leaves and panicles but only of the whole canopy. These spectrometers enable us to collect reflectance spectra for individual organs but at the price of low efficiency with small sampling areas, inconsistent sun-view geometry, and high sensitivity to sample desiccation. Given these limitations, little is known about the spectral properties of rice panicles and their spectral differences from rice leaves under natural light conditions. With the use of imaging spectrometers in the field, the highly efficient collection of spectra for the coexistent leaves and panicles could be achieved.

Recently, near-ground imaging spectroscopy has emerged as a promising sensing technique that can provide us with very high spatial resolution imaging spectroscopy data for examining individual canopy components of crops [25]. With a simple setup of an imaging spectrometer, Zhang et al. [26] collected hyperspectral images of a detached branch tiled on a grey board and examined the shadow effects on 14 vegetation indices (VIs) between SL and SHL. Their findings will be limited for practical canopy-level applications because the light interaction with leaves in a canopy is much more complicated. Canopy-level experiments would be more appropriate for investigating the shadow effects on spectral properties between SL and SHL. A few studies involved the discrimination of SL and SHL pixels [5,7] in hyperspectral images of this kind, but they paid attention to the further analysis of only sunlit pixels and neglected the shaded pixels of the canopy. To date, little is known about how the spectral properties of rice leaves or panicles differ between sunlit and shaded counterparts. The use of very high resolution imaging spectroscopy data opens new opportunities for the determination of such differences over the whole growing season.

The research objectives of this study were to examine the seasonal spectral variation of leaf and panicle components in rice canopies and to determine the difference in spectral properties between SL and SHL over the whole growing season for improved quantification of foliar chlorophyll content. Specifically, we strived to answer the following three questions: (1) What are the spectral differences between leaves and panicles coexistent in rice canopies? (2) Are the sunlit and shaded parts of leaves or panicles separable spectrally? (3) Is there any difference in correlations with foliar chlorophyll content and VIs of SL and SHL?

## 2. Materials and Methods

### 2.1. Study Site and Experimental Design

The paddy field site is located in Rugao, Jiangsu, China (120°19′ E, 32°14′ N). The annual average temperature is 14.6 °C and annual average precipitation is 1055.5 mm, respectively. This rice (*Oryza sativa* L.) experiment encompassed four N fertilization rates (0, 100, 200 and 300 kg·N·ha^−1^) with a row spacing of 30 cm for the minimum and maximum rates and two row spacings (30 cm and 50 cm) for the intermediate rates. Two rice cultivars (Japonica rice: Wuyunjing 24 and Indica rice: Y liangyou 1) were grown in all experiments. A total of 12 plots were grown for the whole study. The individual plot size was 5 m by 6 m.

### 2.2. Acquisition and Preprocessing of Imaging Spectroscopy Data

#### 2.2.1. Image Data Acquisition

**Figure 1:**
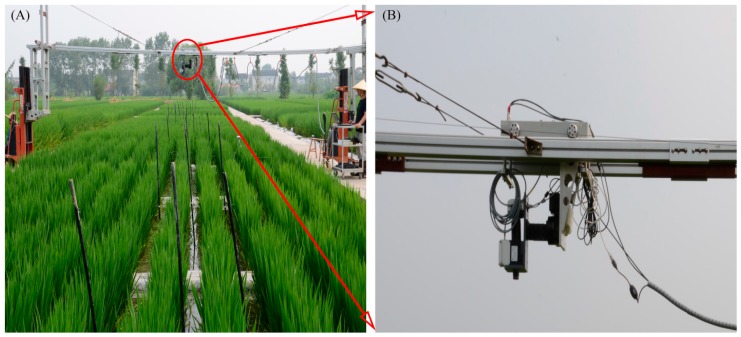
(**A**) Setup of the near-ground hyperspectral imaging system in the rice field and (**B**) onset of the hyperspectral camera in the system.

The hyperspectral images were collected by a pushbroom scanning sensor (V10E-PS, SpecIm, Oulu, Finland) mounted on a platform about 1.2 m above the rice canopies (Figure 1). The sensor recorded data at 520 bands in the visible and near-infrared (NIR) regions (360–1025 nm) with a spectral resolution of 2.8 nm. The spatial resolution of near-nadir viewing (field of view of 42.8°) was approximately 1.3 mm and the swath width was about 90 cm. Hyperspectral images were collected under natural light conditions (Table 1) with the sensor exposure time fixed manually to adapt to brightness variation between scans. Typically, the exposure time for cloudless weather is about 0.2 ms. The imaging system scanned the crops across the row orientation (5 m wide) to complete a scene and a total of 12 image scenes were acquired at each growth stage (Table 1).

#### 2.2.2. Data Preprocessing

**Figure 2:**
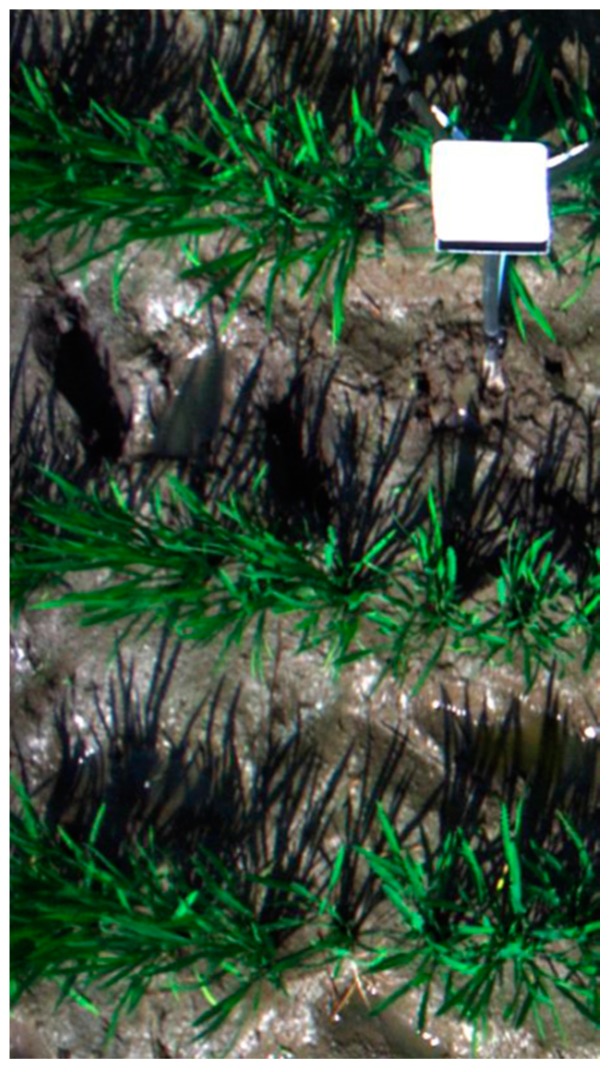
An example true color image cropped from a hyperspectral scene acquired on 20 July 2014.

The raw images were processed with the subtraction of sensor dark current and radiometric correction by the software specVIEW (SpecIm, Oulu, Finland) in the instrument system. The original digital number (*DN*) values were converted to relative reflectance. The calibration equation was as follows [27]:

$$Ref_{\mathrm{target}}=\frac{DN_{\mathrm{target}}-DN_{\mathrm{noise}}}{DN_{\mathrm{panel}}-DN_{\mathrm{noise}}}\times Ref_{\mathrm{panel}}$$

where *DN*_target_, *DN*_noise_ and *DN*_panel_ refer to the *DN* value of target, electronic noise and reference panel, respectively. *Ref*_target_ and *Ref*_panel_ refer to the reflectance value of target and reference panel, respectively. The radiometric calibration process was implemented as suggested in Herrmann et al. [28] by placing a barium sulfate (BaSO_4_) panel as the white reference on the tripod (Figure 2). The relative reflectance data were smoothed using the Minimum Noise Fraction (MNF) transform. The spectral data in the 400–900 nm range were retained due to strong noise in other spectral regions even after smoothing. We built spectral libraries of sunlit and shaded components by choosing regions of interest (ROIs) manually with approximately 80 pixels for each class from 12 images at every growth stage. Specifically, there were 5760 pixels for SL, 5760 pixels for SHL, 1920 pixels for sunlit panicles (SP) and 1920 pixels for shaded panicles (SHP), respectively.

To evaluate the relationship of VIs derived from all pixels of SL or SHL across the whole image with foliar chlorophyll content, we firstly used the enhanced vegetation index (EVI) to create non-vegetation masks as suggested in Pinto et al. [19]. Vegetation pixels were identified with an EVI value of greater than 0.45. Afterwards, we used the established spectral library and the decision tree for discriminating all the pixels of sunlit and shaded canopy components in the images.

### 2.3. Experimental Measurements

**Figure 3:**
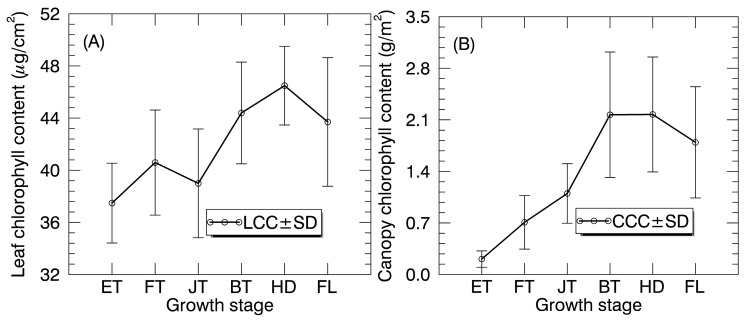
The temporal profiles of (**A**) LCC and (**B**) CCC in paddy rice over the whole growing season. LCC and CCC values are shown as mean ± standard deviation (SD).

The SPAD-502 (Minolta Camera Co., Osaka, Japan) chlorophyll meter was used to take SPAD readings from the three uppermost fully expanded leaves with the mean being the representative value [29]. In particular, three randomly selected plants from each field plot were measured. Three SPAD values per leaf, including one around the midpoint of the leaf blade and two 3 cm apart from the midpoint, were averaged as the mean SPAD value of each leaf [29]. The leaf chlorophyll content (LCC, μg/cm^2^) was calculated from the SPAD readings using the equation built with a subset of samples from this study as shown below:

(1) y=1.4498×x−22.014

where y and x are LCC and SPAD reading, respectively. Leaf area was measured with a leaf area meter LI-3000 (LI-COR, Inc., Lincoln, NE, USA) and divided by the corresponding ground area to determine the leaf area index (LAI, m^2^/m^2^). The canopy chlorophyll content (CCC, g/m^2^) was calculated as the product of LCC and LAI. A summary of the chlorophyll content data was provided in Table 2. Additionally, the temporal profiles of LCC and CCC were presented in Figure 3. LCC and CCC values exhibited a tendency to increase and then decrease in the whole season with the peak being observed at the heading stage for LCC but at booting stage for CCC (Figure 3). Specially, a local minimum of LCC value was found at the jointing stage.

### 2.4. Calculation of VIs and Continuum Removal

We investigated the four VIs including normalized difference vegetation index (NDVI) [30], transformed chlorophyll absorption reflectance index (TCARI) [31], photochemical reflectance index (PRI) [32] and red edge chlorophyll index (CI_Red-edge_) [33], which are specifically related to canopy greenness, pigment content, photosynthetic light use efficiency and chlorophyll content.

(2) $$\mathrm{NDVI}=\left(R_{800}-R_{670}\right)/\left(R_{800}+R_{670}\right)$$

(3) $$\mathrm{TCARI}=3\times \left[\left(R_{700}-R_{670}\right)-0.2\times \left(R_{700}-R_{550}\right)\cdot \left(\frac{R_{700}}{R_{670}}\right)\right]$$

(4) $$\mathrm{PRI}=\left(R_{531}-R_{570}\right)/\left(R_{531}+R_{570}\right)$$

(5) $${\mathrm{CI}}_{\mathrm{Red}-\mathrm{edge}}=\left(R_{800}/R_{720}\right)-1$$

where R_531_, R_550_, R_570_, R_670_, R_700_, R_720_, R_800_ represent the reflectance values at 531, 550, 570, 670, 700, 720, 800 nm, respectively. In order to compare the shapes of absorption features, we applied a method of normalization, called continuum removal [34], to the carotenoid and chlorophyll absorption features in the blue (400–550 nm) and red (550–750 nm) domains, respectively [35,36].

### 2.5. Spectral Matching Analysis

To obtain better quantification merits for the spectral differences between individual canopy components, two commonly used classification methods (i.e., Spectral Angle Mapper (SAM) [37] and Spectral Information Divergence (SID) [38]) were used to investigate the differences in the reflectance spectra or continuum-removed spectra between different canopy components. Additionally, the classification accuracies produced using SAM and SID have also been compared with that produced using the decision tree generated in this study.

## 3. Results

### 3.1. Comparisons of the Reflectance Amplitude and Absorption Feature between Canopy Components

**Figure 4:**
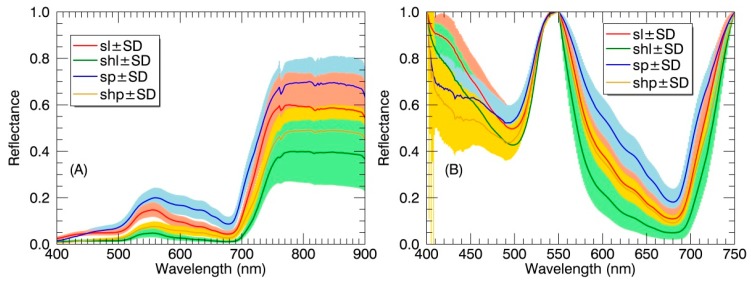
(**A**) The reflectance spectra and (**B**) continuum-removed reflectance spectra of different canopy components in paddy rice over the whole growing season. Spectral values are shown as mean ± standard deviation (SD). SL: sunlit leaf; SHL: shaded leaf; SP: sunlit panicle; SHP: shaded panicle.

Generally, the sunlit components exhibited higher average reflectance values than their shaded counterparts (Figure 4A) at all wavelengths (400–900 nm). In particular, the reflectance values of sunlit components in the visible region (especially in green bands) were systematically higher than those of shaded counterparts, while there were overlaps in the NIR region. Regardless of sunlit or shaded parts, the reflectance of panicles was higher than that of leaves in the visible region except before 450 nm. Unlike reflectance differences, the absorption features were stronger for the shaded components than those for their sunlit counterparts (Figure 4B). In the 400–550 nm range, the absorption feature of panicles differed greatly in shape from that of leaves due to the broadening in the blue region.

As shown in Table 3 and Table 4, the differences between reflectance spectra of individual classes and between the continuum-removed spectra of individual classes were further compared using SAM and SID. Generally, the SAM values (i.e., spectral angles) between panicle and leaves for continuum-removed spectra were higher than those for reflectance spectra. In contrast, this pattern was not observed in the differences of SID values. Specially, the highest SAM and SID values were both found between the spectra of SP and those of SHL.

### 3.2. Seasonal Variation in Reflectance of Leaf and Panicle Components

**Figure 5:**
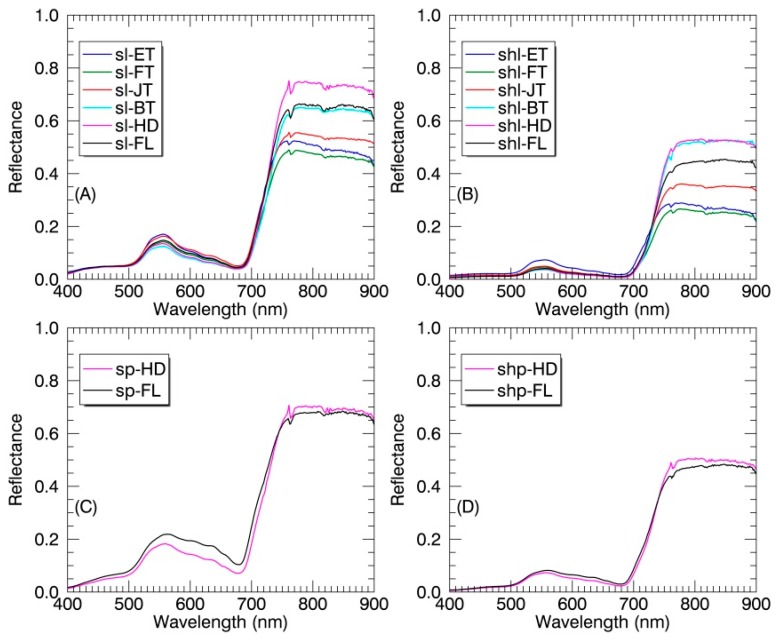
The average reflectance spectra of SL (**A**), SHL (**B**), SP (**C**) and SHP (**D**) in paddy rice at individual growth stages. ET: early tillering stage; FT: fully tillering stage; JT: jointing stage; BT: booting stage; HD: heading stage; FL: filling stage.

Figure 5 shows the average reflectance of individual canopy components in paddy rice over critical growth stages. For both SL and SHL, the reflectance exhibited greater variability between growth stages in the NIR region than in the visible region. The across-stage variability in the NIR region was not observed for either SP or SHP. Specifically, the NIR reflectance of both SL and SHL increased gradually from the early tillering stage to the heading stage and declined after the heading stage (Figure 5A,B). The NIR reflectance of panicles (Figure 5C,D) decreased slightly but exhibited an increase in the visible region, especially for the SP.

### 3.3. Seasonal Variation in VIs of Leaf and Panicle Components

**Figure 6:**
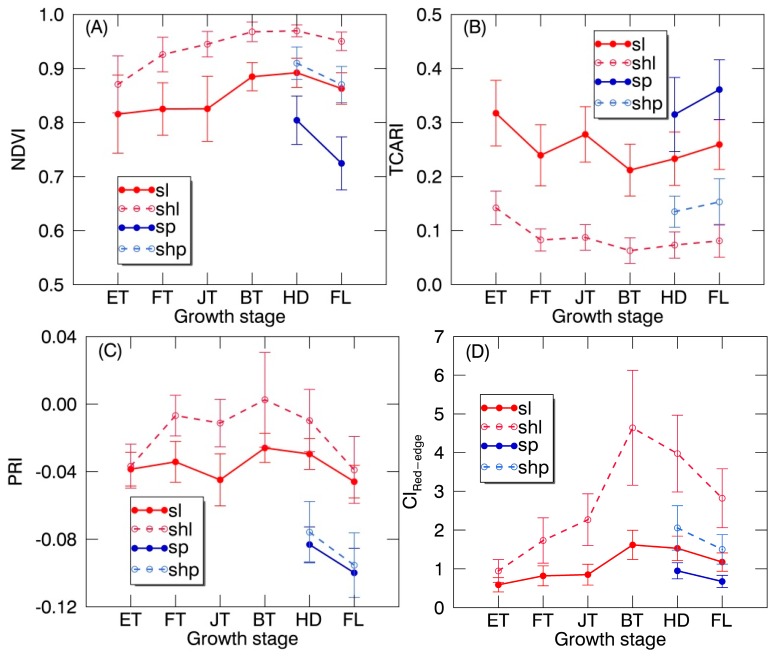
The temporal profile (mean ± standard deviation) of (**A**) normalized difference vegetation index (NDVI), (**B**) transformed chlorophyll absorption reflectance index (TCARI), (**C**) photochemical reflectance index (PRI) and (**D**) red edge chlorophyll index (CI_Red-edge_) derived from regions of interest (ROIs) for SL, SHL, SP and SHP over the whole growing season. ET: early tillering stage; FT: fully tillering stage; JT: jointing stage; BT: booting stage; HD: heading stage; FL: filling stage.

The temporal patterns of PRI and CI_Red-edge_ were generally similar to that of NDVI except the maxima being observed at the booting stage as compared to the heading stage for NDVI (Figure 6). The temporal variation in TCARI across stages was different from that in all the three VIs. Generally, the shaded components exhibited higher values for NDVI, PRI and CI_Red-edge_ but lower values for TCARI than their sunlit counterparts at each stage. In particular, the temporal profiles of TCARI across all stages were clearly separable between sunlit components and their shaded counterparts (Figure 6B). Paired *t*-tests supported that there were significant differences in TCARI for SL vs. SHL (*p* < 0.0001) and SP vs. SHP (*p* < 0.0001). However, the TCARI overlapped between sunlit components (i.e., SL and SP) and also overlapped between shaded components (i.e., SHL and SHP).

**Figure 7:**
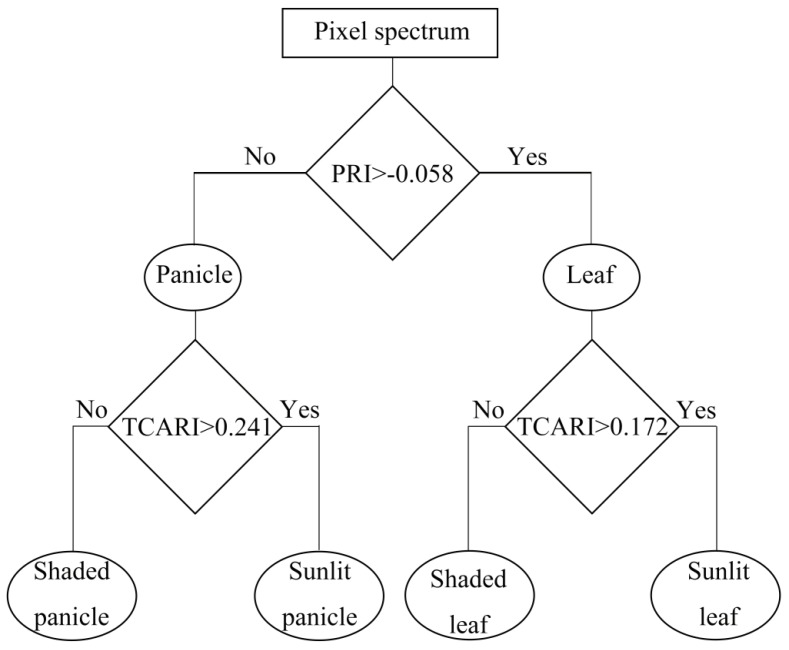
Decision tree for separating four canopy components: SL; SHL; SP; SHP across all growth stages. Each option (yes or no) leads to a condition or to a classification product. The PRI threshold for separating leaves and panicles was determined as their respective mean PRI values of all the ROIs datasets averaged over the whole growing season. In the same way, the TCARI thresholds for separating SL vs. SHL and SP vs. SHP were determined by averaging their respective mean TCARI values.

**Figure 8:**
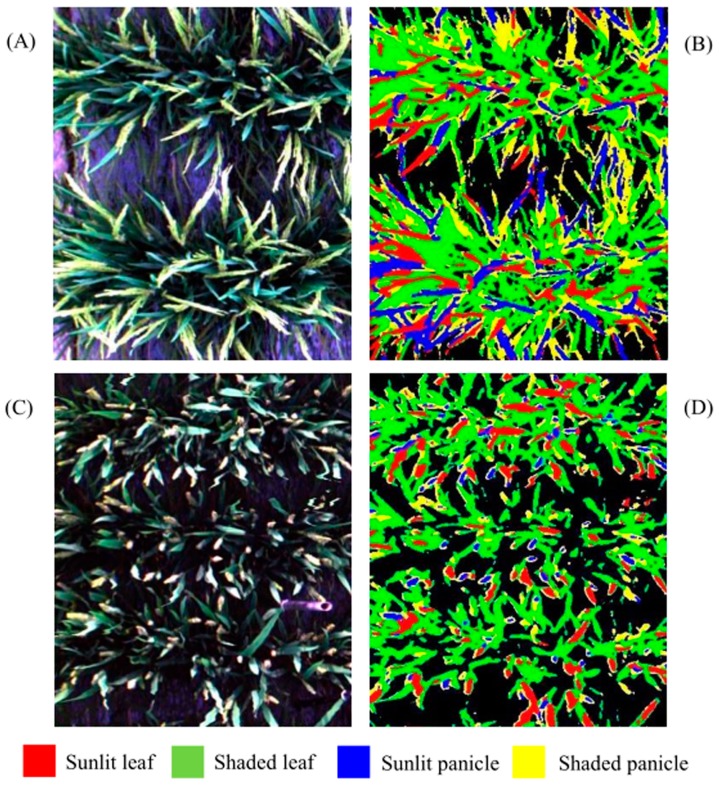
Two true color composites ((**A**) Indica rice; (**C**) Japonica rice) and decision tree classification maps ((**B**) Indica rice; (**D**) Japonica rice) for the rice experiment at the heading stage. The black background in (**B**,**D**) represents non-vegetation pixels.

PRI expressed the least separability between each sunlit component and its shaded counterpart compared to other VIs (Figure 6C), especially at the early tillering stage and the filling stage. However, there were significant differences in PRI between leaves and panicles (*p* < 0.0001) regardless of sunlit or shaded ones. By applying PRI and TCARI thresholds derived from all the ROIs datasets of individual classes throughout the whole growing season at two sequential steps, a simple decision tree was constructed as shown in Figure 7 to classify the canopy components. The ten-fold cross-validation demonstrates that the images could be classified into four classes including SL, SHL, SP and SHP with an overall accuracy of 90.56% (Figure 8). This accuracy was substantially higher than those produced using the classification method of SAM (overall accuracy of 58.14% and 64.88% for using reflectance spectra and continuum-removed spectra, respectively) and SID (overall accuracy of 72.67% and 72.01% for using reflectance spectra and continuum-removed spectra, respectively).

### 3.4. Relationships between VIs and Foliar Chlorophyll Content for SL and SHL

As shown in Table 5, most VIs of SHL displayed stronger relationships with LCC and with CCC than those of SL. In particular, the TCARI of SHL exhibited much higher correlations with LCC (R^2^ = 0.62, *p* < 0.0001) and CCC (R^2^ = 0.75, *p* < 0.0001) than that of SL (LCC: R^2^ = 0.26, *p* < 0.0001; CCC: R^2^ = 0.25, *p* < 0.0001). In contrast, the CI_Red-edge_ exhibited the strongest correlations among all VIs with CCC for both SL (R^2^ = 0.84, *p* < 0.0001) and SHL (R^2^ = 0.90, *p* < 0.0001). Greater contrast in R^2^ between SL and SHL was found for TCARI and PRI than for NDVI and CI_Red-edge_.

**Figure 9:**
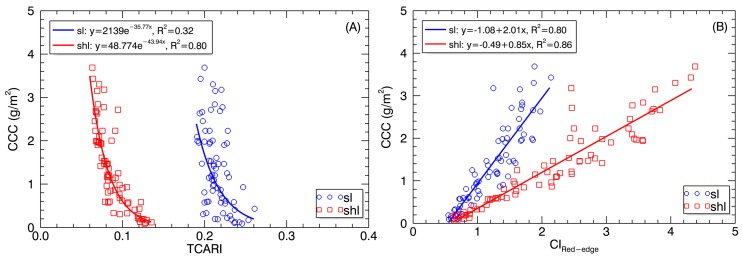
Best-fit relationships of (**A**) TCARI and (**B**) CI_Red-edge_ derived from SL and SHL pixels with CCC.

**Figure 10:**
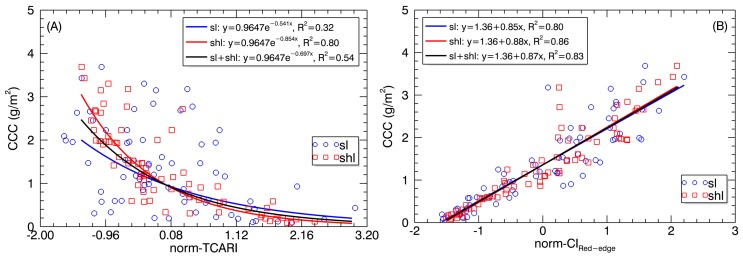
Relationships of the (**A**) normalized TCARI (norm-TCARI) and (**B**) normalized CI_Red-edge_ (norm-CI_Red-edge_) derived from SL and SHL pixels with canopy chlorophyll content.

Figure 9 shows the regression plots with two representative VIs that respectively exhibited the most contrasting relationships (for TCARI) or the closest relationships (for CI_Red-edge_) with CCC between SL and SHL. The regression models for the TCARI were apart between SL and SHL as a result of systematic offset in TCARI, but the models for the CI_Red-edge_ differed significantly in slope. After z-score normalization of TCARI values for SL and SHL individually (Table 6), the TCARI–CCC exponential models were both closer to the model generated by combining SL and SHL. The R^2^ value for the combined data (R^2^ = 0.54) was higher than that for SL (R^2^ = 0.32) but lower than that for SHL (R^2^ = 0.80) (Figure 10A). With normalized CI_Red-edge_ data, the CI_Red-edge_–CCC linear models for SL and SHL converged to the model for combining SL and SHL with a comparable R^2^ value (Figure 10B).

## 4. Discussion

### 4.1. Differences in the Reflectance Amplitude and Pigment Absorption Features between Canopy Components

Our results demonstrated that the sunlit components exhibited higher reflectance values than their shaded counterparts (Figure 4A and Figure 5A,B). The lower reflectance amplitude observed for shaded components (Figure 4A) is mainly due to the weaker irradiance composed mainly of diffuse light and subsequently lower intensity of the reflected signal [18]. This pattern was in agreement with the previous studies of shadow effects on reflectance at the canopy level [2] and the leaf level [26]. The shaded components showed less than one percent reflectance in the visible region and thus appeared dark to human eyes [26]. The NIR reflectance of shaded leaves was nearly one and one-half times less than that of sunlit leaves at each growth stage instead of five times less than that of sunlit leaves found in Zhang et al. [26]. This might be due to the within-canopy multi-scattering and greater reflectance for shaded leaves at the canopy level than at the leaf level. With regard to the shadow effects on the absorption features, the stronger absorption features for shaded components (Figure 4B) is caused by more light absorbance [39] and higher light use efficiency at the leaf level [20]. Moreover, for the canopy level, multiple scattering of photons within rice canopies contributed to the stronger apparent absorption by shaded components, especially in the red region [34,40].

**Figure 11:**
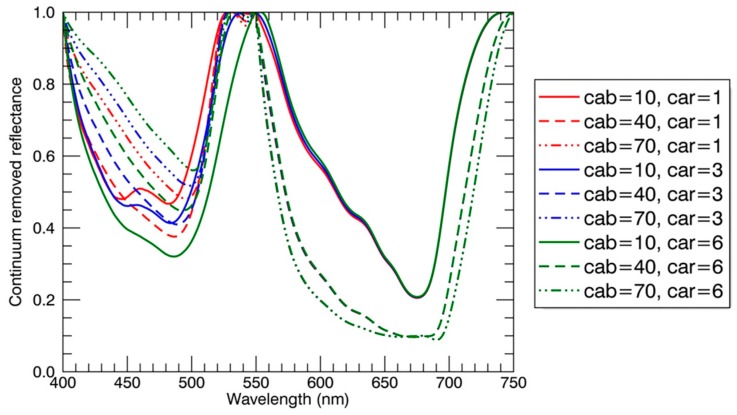
The continuum removed reflectance spectra derived from the simulated reflectance spectra with the PROSAIL model. Fixed parameters for the PROSAIL model were: equivalent water thickness = 0.018 cm, dry matter content = 0.004 g/cm^2^, leaf structure parameter = 1.55, average leaf angle = 45°, LAI = 2.0, hot spot = 0.15, solar zenith angle = 30°, view zenith angle = 0°, relative azimuth angle = 0°, diffuse/direct radiation = 10. Varying parameters for the PROSAIL model were: chlorophyll content (Cab) = 10 μg/cm^2^, 40 μg/cm^2^, 70 μg/cm^2^; carotenoid content (Car, μg/cm^2^) = 1 μg/cm^2^, 3 μg/cm^2^, 6 μg/cm^2^.

Regardless of sunlit or shaded parts, the reflectance of panicles was significantly higher than that of leaves in the visible region (Figure 4A). Moreover, the shape of absorption features in the blue region for panicles differed greatly from that for leaves (Figure 4B). The above two patterns could be explained by the much lower chlorophyll and carotenoid contents in rice panicle (nearly 12 times less) than that in rice leaf [35]. In particular, the double-peak absorption feature in the blue region for panicles (Figure 4B) may be attributed to the domination by carotenoids over chlorophylls [41]. To test the effects of variation in chlorophyll and carotenoid content on the absorption features in the blue region, we used the commonly used radiative transfer model PROSAIL [42,43] and the continuum removal method to generate simulated continuum-removed reflectance. Figure 11 shows that the double-peak feature in the blue region is most significant at the lowest chlorophyll and carotenoids contents. This could probably explain the unique shape of absorption features in the blue region for rice panicles. Additionally, the unique shape of absorption features in the blue region for panicles could be the main driver of the higher spectral angles between panicles and leaves (Table 3) and the better classification performance while using the continuum-removed spectra instead of reflectance spectra as the reference spectra in the SAM classification, which extracts geometric features between two spectra as compared to measuring the discrepancy of probabilistic behaviors between two spectra in the SID classification [44].

### 4.2. Spectral Separation between Canopy Components

The temporal profiles of VIs observed for rice canopies showed a clear dependency on the shadow effects (Figure 6). Specifically, the TCARI between sunlit and shaded counterparts were clearly distinguishable for all stages (Figure 6B). Given that the TCARI was originally developed as a strong indicator of crop chlorophyll content and had been widely used for such a purpose in the community [31], the clear separation of SL and SP from SHL and SHP could be due to their differences in the strength of apparent chlorophyll absorption [34,45,46]. Meanwhile, utilizing green light around 550 nm in TCARI might further enhance this separability (Figure 4A).

The PRI was developed to track the reversible photoprotective responses of the xanthophyll pigment cycle and used as an indicator of LUE in many studies [18,20,32]. Our results confirmed that SHL exhibited higher PRI values and accordingly higher LUE than SL that were more likely to experience excessive light stress and to have lower LUE [20,21,47,48]. Although the PRI represented less significant differences between sunlit and shaded components than other VIs, it exhibited the most pronounced separability between leaves and panicles regardless of being sunlit and shaded (Figure 6C). Compared to leaves, the systematically lower LUE for panicles could be the main driver of the lower PRI values due to the substantially less efficient photosynthesis [49].

### 4.3. Gain from the Overlooked Pixels of Shaded Leaves

Previous studies often focused on the sunlit pixels for classification or chemistry quantification purposes, but they neglected or weakened the importance of shaded pixels due to the insufficient spatial resolution or signal-noise-ratio in the shaded crown pixels [5,7,15,50]. Taking the estimation of chlorophyll content as an example, we found higher correlations between most VIs and chlorophyll content at leaf and canopy levels in SHL than in SL (Table 5). The main reason could be the fact that multiple scattering among SHL leads to extended optical length with even weaker signal intensity [51,52] and enhances the apparent absorption features of leaves [34,40,42]. The signal from SHL was not weak since a larger number of SHL pixels existed in the canopy images. Specifically, the average sunlit/shaded foliage ratios of pixel numbers extracted from individual stages were all less than 0.5, except for the early tillering stage with an average ratio of above 1.5.

The stability in correlation with chlorophyll contents over shaded and sunlit pixels varied with VIs. The main reason for the most contrasting relationships for TCARI might be because SL were much easier to exhibit saturate absorption than SHL in the red absorption region, which was more effectively characterized in TCARI than other VIs (e.g., NDVI). Thus, caution should be exercised about the choice of VIs and the use of sunlit or shaded pixels to estimate the CCC. If TCARI and PRI are used, they should be derived from shaded pixels for better estimation of CCC.

In contrast, using the CI_Red-edge_ data from sunlit pixels as usual would not result in a significant loss in accuracy, but would lead to a different model compared to shaded pixels (Figure 9B). Fortunately, a normalization of the CI_Red-edge_ data from shaded and sunlit pixels individually could remove this model difference (Figure 10B). This might be explained by the fact that the CI_Red-edge_ exhibited a strong linear relationship between SL and SHL (R^2^ = 0.94, *p* < 0.0001), which was stronger than relationships with other VIs (R^2^ = 0.51–0.76, *p* < 0.0001). The strong linear relationships of CI_Red-edge_ to LCC or CCC for both SL and SHL might be attributed to the sensitivity of red edge bands to chlorophyll and the deep penetration of red edge light into leaves and canopies [53], which is four- to six-fold higher than that of blue and red light [33]. Besides the stability between sunlit and shaded pixels, CI_Red-edge_ exhibited superior performance in the linear regression models that are common for sunlit and shaded leaf pixels after a normalization and do not suffer from saturation as was the case for other VIs such as TCARI.

### 4.4. Implications for Future Work

Shadows were found to exhibit significant effects on the spectral properties of leaves and their relationships to chlorophyll content. Nevertheless, the plant physiological responses to changing light conditions could complicate quantitative assessments of shadow effects when using observational data [18]. For example, the vertical distribution of nitrogen or chlorophyll is the transference of nitrogen to the growing center (e.g., the new leaves emerged from the sheath) and also an adaptation to the light distribution within canopies [54]. The spectral differences between sunlit-upper layer leaves and shaded-lower layer leaves likely depend not only on the vertical variation of illumination conditions but also on the non-uniform vertical distribution of nitrogen or chlorophyll. Our future work would be directed to the use of multi-angle viewing imaging spectroscopy data, which could be more effective than only the nadir viewing data. A limitation of this study is the extremely high spatial resolution of hyperspectral images, which is unavailable from current satellite data. However, it is possible to make these image measurements to a larger area from a low-altitude unmanned aerial vehicle (UAV) system equipped with a hyperspectral camera [8].

## 5. Conclusions

We report on the use of near-ground hyperspectral imaging data to investigate the spectral properties of rice leaf and panicle components under the natural illumination conditions for better understanding the within-canopy spectral variation of rice. The shaded components exhibited lower reflectance amplitude but stronger absorption features than their sunlit counterparts. Specifically, unique shapes of double-peak absorption features in the blue region were observed for panicle spectra. Among the examined VIs, the TCARI exhibited significant differences between sunlit and shaded components regardless of leaves and panicles. For either sunlit or shaded components, the PRI exhibited significant differences between leaves and panicles. In addition, the significant differences observed for these two indices occurred over the whole growing season.

While correlated to CCC, the VIs for SL and SHL pixels showed diverse patterns. Stronger correlations of CCC with VIs including PRI and TCARI were observed for SHL pixels than for SL pixels. The correlations with CI_Red-edge_ were even stronger but were not significantly different between SL and SHL pixels. These results represent significant advances in the spectral properties of overlooked shaded pixels within the canopy and demonstrate the potential for improved estimation of CCC with high resolution imaging spectroscopy data.

This research provides useful information for improving our understanding of the spectral variation within rice canopies and the shadow effect on the spectral properties of rice leaves. The study adds new knowledge of leaf and panicle spectral properties under sunlit and shaded conditions to the current crop canopy sensing community. It is also beneficial for resolving the photosynthetic signals of sunlit and shaded leaves and has great potential for reducing the uncertainties in the estimation of canopy chemistry for individual plants.

## Figures and Tables

**Table 1:** Summary of image acquisition conditions for the rice experiment.

Date	Time (GMT+8)	Range of Solar Zenith Angle	Growth Stage
8 July 2014	09:50–14:50	31.1°–37.9°	Early tillering
20 July 2014	10:00–15:20	30.1°–44.7°	Fully tillering
4 August 2014	10:05–15:20	30.9°–46.3°	Jointing
20 August 2014	10:00–15:10	34.2°–47.1°	Booting
3 September 2014	10:10–15:30	35.1°–54.8°	Heading
20 September 2014	09:40–15:00	43.8°–54.0°	Filling

**Table 2:** Statistics of leaf and canopy chlorophyll content data for the experiment. Min: minimum value; Max: maximum value; Mean: mean value; SD: standard deviation; CV: coefficient of variation.

Chlorophyll Parameters	Min	Max	Mean	SD	CV (%)
Leaf chlorophyll content (LCC) (μg/cm^2^)	30.51	48.80	41.94	4.94	11.77
Canopy chlorophyll content (CCC) (g/m^2^)	0.09	3.69	1.36	0.95	69.96

**Table 3:** The averages of spectral angle mapper (SAM) values (unit: radians) for all spectra of each class (No. = 5760) as compared with mean reflectance spectrum/mean continuum-removed spectrum of individual classes.

Averages of SAM Values	Mean Spectrum of SL	Mean Spectrum of SHL	Mean Spectrum of SP	Mean Spectrum of SHP
SL	0.07/0.08	0.15/0.15	0.11/0.20	0.11/0.16
SHL	0.14/0.18	0.08/0.12	0.20/**0.31**	0.10/**0.21**
SP	0.10/**0.19**	0.21/**0.29**	0.06/0.08	0.15/0.16
SHP	0.10/0.17	0.07/0.20	0.15/0.18	0.05/0.10

The highest correlations in each column are highlighted in bold.

**Table 4:** The averages of spectral information divergence (SID) values for all spectra of each class (No. = 5760) as compared with mean reflectance spectrum/mean continuum-removed spectrum of individual classes.

Averages of SID Values	Mean Spectrum of SL	Mean Spectrum of SHL	Mean Spectrum of SP	Mean Spectrum of SHP
SL	0.01/0.01	0.06/0.02	0.03/0.03	0.02/0.02
SHL	**0.06**/0.03	0.02/0.01	**0.13**/0.08	**0.04**/**0.04**
SP	0.03/0.03	**0.13**/0.07	0.00/0.01	**0.04**/0.02
SHP	0.01/0.02	0.03/0.04	0.04/0.02	0.01/0.01

The highest correlations in each column are highlighted in bold.

**Table 5:** Squared correlation coefficients (R^2^) from Spearman correlation for VIs derived from SL and SHL pixels in relation to LCC and CCC.

VIs	LCC	CCC
NDVI_sl	0.45 **	0.63 **
NDVI_shl	0.58 **	0.81 **
TCARI_sl	0.26 **	0.25 **
TCARI_shl	0.62 **	0.75 **
PRI_sl	0.08	0.11 *
PRI_shl	0.29 **	0.46 **
CI_Red-edge__sl	0.54 **	0.84 **
CI_Red-edge__shl	0.61 **	0.90 **

* *p* < 0.01; ** *p* < 0.0001. The highest correlations are highlighted in bold.

**Table 6:** Coefficient of determination (R^2^) for the relationships between z-score normalized VIs derived from SL and SHL pixels and CCC.

VIs	SL		SHL		Combined	
	Linear Fit	Exponential Fit	Linear Fit	Exponential Fit	Linear Fit	Exponential Fit
NDVI	0.55	0.63	0.62	0.87	0.58	0.75
TCARI	0.23	0.32	0.63	0.80	0.41	0.54
PRI	0.11	0.08	0.50	0.40	0.27	0.21
CI_Red-edge_	0.80	0.78	0.86	0.81	0.83	0.80

## Abbreviations

The following abbreviations are used in this manuscript:

SL	Sunlit Leaf
SHL	Shaded Leaf
SP	Sunlit Panicle
SHP	Shaded Panicle
LUE	Light Use Efficiency
VIs	Vegetation Indices
NIR	Near-Infrared
ROIs	Regions Of Interest
LCC	Leaf chlorophyll content
CCC	Canopy chlorophyll content
NDVI	Normalized Difference Vegetation Index
TCARI	Transformed Chlorophyll Absorption Reflectance Index
PRI	Photochemical Reflectance Index
CI_Rededge_	Red Edge Chlorophyll Index
